# Testing Emotional Vulnerability to Threat in Adults Using a Virtual Reality Paradigm of Fear Associated With Autonomic Variables

**DOI:** 10.3389/fpsyt.2022.860447

**Published:** 2022-04-01

**Authors:** Marcus L. Brandão, Manoel Jorge Nobre, Ruth Estevão

**Affiliations:** ^1^Instituto de Neurociências e Comportamento, São Paulo, Brazil; ^2^NAP-USP-Neurobiology of Emotions Research Centre (NuPNE), Ribeirão Preto Medical School, University of São Paulo (FMRP-USP), São Paulo, Brazil

**Keywords:** fear, anxiety, emotional resilience, aversive stimuli, defense reaction

## Abstract

Fear and anxiety are generally assessed as responses of prey to high or low levels of threatening environments, fear-conditioned or unconditioned stimuli, or the intensity and distance between predator and prey. Depending on whether a threat is close to or distant from the individual, the individual exhibits specific behaviors, such as being quiet (freezing in animals) if the threat is distant or fleeing if the threat is close. In a seminal paper in 2007, Dean Mobbs developed an active prevention virtual reality paradigm (VRP) to study a threat’s spatial imminence using finger shocks. In the present study, we used a modified VRP with a distinctive feature, namely a dynamic threat-of-loud noise paradigm. The results showed a significant reduction in the number of times the subjects were captured in the high predator phase (85 dB) vs. control phases, suggesting that the participants were motivated to avoid the high predator. Concomitant with avoidance behavior, a decrease in respiratory rate and an increase in heart rate characterized the defense reaction. These results demonstrate behavioral and autonomic effects of threat intensity in volunteers during a VRP, revealing a profile of defense reaction that reflects the individual emotional susceptibility to the development of anxiety.

## Introduction

Behavioral studies of defense reactions indicate that an organism’s defensive strategies are diverse and highly organized [for review, see (1–3)]. Indeed, fear is more than a simple aversive event—it is a mental state that is induced by a perceived threat that causes behavioral and related autonomic changes that constitute an adaptive response of the organism that is directed toward survival (4, 5). A general framework has emerged from studies of predatory threat in rodents (6–8). Strategies range from the global behavioral organization of ascending levels of threat that is associated with pre-encounter, post-encounter, and circa-strike, or fight/flight at the encounter with the predator (7–9). Neurobiologists who study fear/anxiety call this the “threat imminence continuum,” which indicates that distinct threat states change, depending on whether the threat context is on or off. The threat imminence continuum encompasses three core phases. In the pre-encounter phase although the presence of threat is not detectable there is a threat context characterized by an increase in vigilance and arousal. The post-encounter phase consists of a predator that starts to chase the aware prey with the intention of capture, resulting in the prey either freezing, escaping, or fighting if the threat is inescapable (3, 7, 8, 10–13). This phase is evoked by defensive motivational circuits that are triggered in the presence of a threat, resulting in a set of survival strategies that are optimized to escape predators (12, 13). The circa-strike phase is when the threat makes contact. Depending on the threat level and proximity, fight/flight behaviors may be triggered at any point of the threat imminence continuum. The concept of “predatory imminence” has been considered as the psychological distance from the predator that is determined by physical, temporal, and probabilistic closeness to contact with the threat (8–11). The reaction is resistant to contextual shifts and generally associated with the memory of the aversive experience (14, 15).

The protocols of the threat imminence continuum are complex and difficult to perform in the laboratory. Classic paradigms, such as fear conditioning, fail to elicit the full range of defensive behaviors and have ambiguous profiles. In the present study, we sought to address these problems by using a virtual reality paradigm (VRP) that was modified from the paradigm developed by Mobbs (10), which explicitly models different levels of predatory threat.

Evidence in the literature indicates limitations of measuring emotions through self-reports or interviews (16–18). Therefore, the present study sought to identify physiological markers that correspond to different states of fear. Breathing is strongly modulated by emotional state and is an important regulator of fear behavior (19–22). We focused on how respiration changes and how it is coupled to blood oxygen levels during different kinds of fear. Finally, we translated to humans reliable data from laboratory studies that investigated the brain processing of threatening situations (3), particularly acoustic signals of an aversive nature. We discuss these states and autonomic variables to make a crucial distinction between different types of fear and implications of this distinction for definitions of mental disorders that are used in psychiatry today.

## Materials and Methods

The experiments were conducted at the Instituto de Neurociencias e Comportamento (INeC) in Ribeirão Preto, São Paulo, Brazil.

### Subjects

The sample comprised 24 subjects (12 women, 12 men) with no history of Diagnostic and Statistical Manual of Mental Disorders, 4th edition, diagnoses of mental disorders. Two subjects were excluded from the data analysis due to technical problems with the equipment. The present study reports unpublished data obtained from volunteers interested in participating in a study that aims to evaluate the individual emotional susceptibility to aversive stimulation during an avoidance/escape virtual application together with measurements of heart rate, respiratory rate, and SpO_2_ levels. The subject filled out a questionnaire and passed by an interview with a psychiatrist to evaluate whether the inclusion criteria for participation in the study were met. The inclusion criteria were the following: (i) 20–60 years old, (ii) no current psychotropic medications, and (iii) no evidence of any organic mental disorder, suicidality, schizophrenia, alcohol or drug dependence, cardiovascular disease, pulmonary disease, epilepsy, or pregnancy. The mean age of the subjects was 48.50 years (standard deviation = 12.72 years). Additional demographic data can be found in Supplementary Table 1.

The study was approved by the Institutional Review Board of the University of São Paulo. All subjects signed an informed consent form before enrollment. Two subjects were excluded from the analysis of results due to equipment problems while recording physiological measures.

### Study Design

Institutional review board approval was obtained for this study (no. 51983721.0.0000.5407). Subjects who agreed to participate in the study were evaluated and enrolled in the study. All subjects were informed about the study by someone from the research team. Subjects with previously documented psychiatric disorders, who were actively using psychiatric medications (e.g., anxiolytics, antidepressants, and antipsychotics) were not included. No medication was given to the subjects during any part of the study. Before starting data acquisition, each subject was encouraged to clarify any concerns because they would not be allowed to talk and had to remain seated during the experiment. Following completion of the questionnaire and interview for each subject, a pulse oximetry probe was placed on the left index finger to measure heart rate and SpO_2_ blood saturation, and a belt was lightly fastened to the thorax to measure respiratory rate. The VRP software was developed by one of the authors of the present study using Python 3.9 software.^1^ Simultaneous physiological measurements with the five phases of the VRP were performed using an interface that was programmed to monitor vital signs (Insight Instruments, Ribeirão Preto, São Paulo, Brazil). Additional details are provided in Supplementary Material.

### Virtual Reality Paradigm

#### Procedure

**FIGURE 1:**
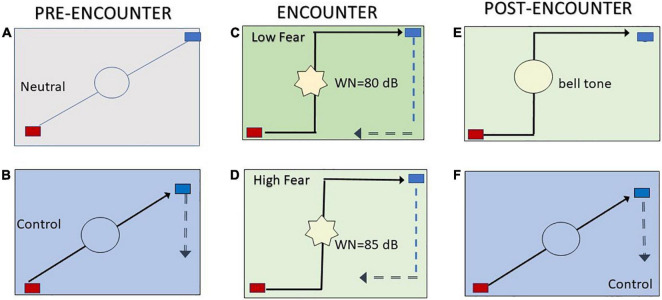
Virtual predator and prey paradigm. Subjects were presented with a two-dimensional open field that contained a 25 cm × 20 cm rectangle. Each of the five blocks of the task began with the subject moving a blue rectangle to (i) avoid the AI predator or (ii) escape the predator by going outside the open field. The experimental condition commenced with a familiarization or neutral phase **(A)**, in which a preprogrammed artificial intelligence (AI) red rectangle appeared at the upper left side of the open field. The AI rectangle was presented for 60 s and programmed to wander the open field randomly and indiscriminately. During this period, the subjects could move a blue rectangle that was in the upper corner of the open field. Each time the red and blue rectangles collided (i.e., encounter or capture), a bell tone (0.5 s) was emitted. Each time the dark blue rectangle went outside the open field (i.e., escape response), a beep tone was emitted. During this period, all aspects of the paradigm were presented to the subject, who was allowed to train to manipulate the dark blue rectangle that represented the “prey” that was placed next to the predator at the upper-left side of the open field. Next, a similar phase **(B)** (phase 1, control) began, with the only difference that the gray background was replaced with a light blue background. Afterward, the AI neutral changed into an AI predator that pursued the prey in two phases. In phase 2 **(C)**, subjects received white noise (80 dB) each time the AI predator captured them (AI low predator). In phase 3 **(D)**, a loud white noise (85 dB) was emitted each time the predator encountered the prey (AI high predator). The change from AI neutral to AI predator was signaled by a green background in phase 2 and a light green background in phase 3. The subjects were not informed that the length of trials varied or given any indication of how much time had elapsed in each trial. Each phase was spaced by a 5 s pause that was indicated by a frozen screen. Next, the post-encounter phase **(E)** was like the previous phase, but the white noise was replaced with a bell tone. For comparison purposes, a control block was included at the end of the session **(F)**. An example of the behavioral responses in the open field during the VRP application developed by this Institute of neuroscience and Behavior (INeC) and the concomitant autonomic responses recorded by the software developed by Insight Medical Instruments is also shown in Supplementary Figure 2.

The subjects were volunteers working in private social care institutions in Ribeirão Preto, São Paulo, Brazil. They were approached in the waiting area approximately 20 min before the test. The subjects were informed about all aspects of the study. Each subject was then asked to answer a few demographic questions and complete a questionnaire. After completing the questionnaire, the subject was taken to the VRP setting. A VRP-compatible belt was fastened on the mid-thorax of the subject to measure respiratory rate during the test. Once all sensors were placed on the subject, the correct operation of the sensors was verified, and physiological data were recorded continuously and simultaneously throughout the VRP session. The session consisted of five phases (see below). Figure 1 summarizes the study design of the behavioral experiment.

The subjects were given instructions about the tasks and received a brief demonstration of how to perform each task. They were asked to be as quiet as possible, making only movements necessary to perform the experimental tasks. The activities were conducted in the same order and had the same duration for each participant subject. After the session, the subjects returned to the waiting area. The subjects were individually debriefed about the study before they saw other participants. None of the subjects left the session prematurely. All subjects were right-handed. Therefore, the sensors were placed on their left hand, enabling them to use their right hand to perform the experimental tasks.

#### Test Session

The paradigm involved the subjects’ trying to avoid a “virtual predator” that could chase, capture, and induce high fear [loud white noise (85 dB): high predator] or low fear [moderate white noise (80 dB): low predator]. A difference of 5 dB between two white noises is noticeable by the humane ear. The experimenters could assess this when they were setting the experimental conditions up in the absence of the subjects. The stress-inducing protocol was composed of five main activities: neutral (familiarization) task, non-stress task (control, no fear stimuli), two stress tasks (low and high predators), and post-stress task. A final condition in which no aversive stimulation was delivered was also added. Each task lasted 60 s with a 5-s intertrial interval.

#### Description of the Virtual Reality Paradigm

Using a modified VRP based on Mobbs et al. (10), the distinction between actual and potential threats was proposed to capture the difference between fear and anxiety. Subjects were presented with a two-dimensional open field that was contained a 22 cm × 24 cm rectangle of walls. The VRP commenced with a “neutral or familiarization phase” (gray background) where a red preprogrammed artificial intelligence (AI) rectangle appeared at the upper-left side of the open field. A blue rectangle that represented the prey was under the control of the subject through movements of the keyboard arrow keys. The subject could move the blue rectangle that represented the prey up, down, left, or right to avoid the AI low or high predator and move outside the open field to escape the predator. The AI red rectangle was programmed to wander around the open field indiscriminately for 60 s. During this time, the subjects were also informed of the amount of aversive stimulation they would receive if the AI predator captured them in the subsequent fear (low and high) phases. Next, during the pre-encounter session (control, blue background), encounter session (low fear, green background), encounter session (high fear, light green background), and post-encounter session (no white noise, light green background), a beep tone was produced each time the “prey” went outside the open field. During each phase, the number of encounters between predator and prey and the number of escapes (going outside the margins of the open field) were recorded. In the control and post-encounter phases (without aversive stimulation), a bell tone was produced each time the “predator” and “prey” collided. The entire session lasted approximately 6 min. White noise was delivered via two speakers only when there was contact with the AI predator. The collision triggered the application of the bell sound or the noise for 0.5 s. The post-encounter phase was a repetition of stress tasks, but a bell tone replaced the aversive stimuli (white noise). This session also served as a control for the “nocebo effect”. The autonomic variables were concomitantly recorded with each behavioral session. A 5 s intertrial black panel separates each phase. The schedule of the overall experiment is also shown in Supplementary Figure 2.

### Device and Sensors

#### Respiratory Rate Sensor

This was a sensor that was attached to a belt that was fastened to the thorax of the subject to measure breathing cycles per minute (respiratory rate). Signals that were detected by the sensor were sent to an interface that was connected to a computer for later analysis.

#### Heart Rate and Oxygen Saturation Measurements

A VRP-compatible pulse oximetry probe was placed on the left index finger of each participant before they started the experimental session. All participants were monitored using a pulse oximeter. We recorded oxygen saturation levels during all phases of the VRP procedures. All measurements were performed in the same room where the experiments were conducted. SpO_2_, respiratory rate, and heart rate were recorded in Excel spreadsheets through a platform for acquiring physiological signals (Insight Equipment, Ribeirão Preto, São Paulo, Brazil) that was coupled to a computer through a local network. Before beginning the reading (before the test), approximately 2 min was allowed to elapse for the oximeter to stabilize.

#### Sound Library

The computer tone and bell tone were downloaded from http://www.freesoundslibrary.com. The white noises (low and high fear noises) were downloaded from the NCH software library. The software license was obtained under license registration ID: 5588311 (NCH software, Canberra, Australia). Two loudspeakers (X-Cell, São Paulo, Brazil) below the screen and 28 cm from the subject were used to deliver the acoustic white noises (80 and 85 dB for the low and high fear conditions, respectively), which were measured by a digital sound level meter (KP 8015, Knup, São Paulo, Brazil).^2^

### Statistical Analysis

We analyzed changes in behavioral responses (avoidance and escape), autonomic parameters (respiratory rate and heart rate), and SpO_2_ levels during the VRP across the five phases of the test (control, moderate and loud noises, post-encounter, and final control). Repeated measures (RM) one-way ANOVA followed by Tukey’s multiple comparisons test was performed using GraphPad Prism version 9.0.0 for Windows, GraphPad Software, San Diego, California, United States.^3^ The number of encounters (collisions) between predator and prey images, escape responses, respiratory rate, heart rate, and SpO_2_ levels were the dependent variables during the five test phases. Whenever significant overall F values were obtained with the repeated measures one-way ANOVA applied on each variable, *post hoc* pairwise comparisons were conducted with the Tukey test, which compares every mean with every other mean and allows for the corrected *P*-value for multiple comparisons. *p* < 0.05 were considered statistically significant. To assess the strength of the prediction of the VRP based on collisions, correlations were determined between the number of encounters during the high fear conditions and respiratory and heart rates using Pearson’s correlation coefficients. Although non-significant these analyses were also conducted with the data recorded during the low fear condition (Supplementary Material).

## Results

**FIGURE 2:**
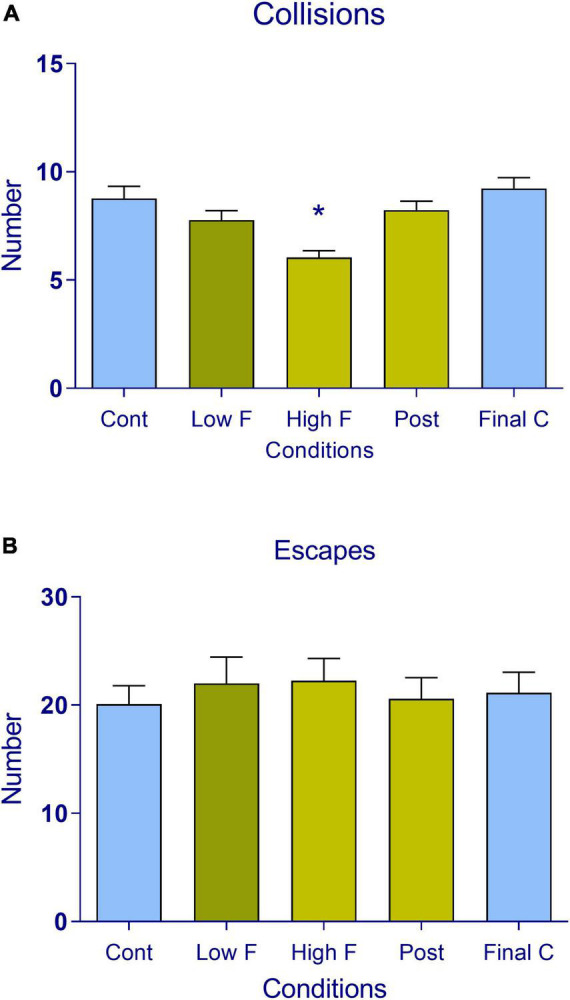
Number of **(A)** collisions (encounters) of the prey/predator and **(B)** escape responses recorded in subjects during the five phases of the virtual reality task (control, low fear, high fear, post-encounter, and final control). The data are expressed as mean + SEM. *n* = 22 across the five conditions (control, low fear, high fear, post-encounter, and final control). *p < 0.05, compared with control.

The responses of subjects while they performed an active avoidance loud noise during prey/predator sessions in control sessions are illustrated in Figure 1. RM one-way ANOVA revealed a significant reduction in the number of times the subjects were captured during the dynamic threat-of-loud noise [*F*(4, 84) = 10.14, *p* < 0.05]. This finding was confirmed by the Tukey *post hoc* analysis, which revealed decreases in collisions in the high predator condition in relation to all other conditions (*p* < 0.05) (Figure 2A). The same analysis of escape behavior was performed in all five conditions of the VRP (Figure 2B), revealing no differences in the number of escapes among all phases of the test [*F*(4, 84) = 0.72, *p* > 0.05]. Thus, the number of escape responses did not change across phases using 80 and 85 dB. Thus, with these noise intensities, avoidance behavior prevails.

**FIGURE 3:**
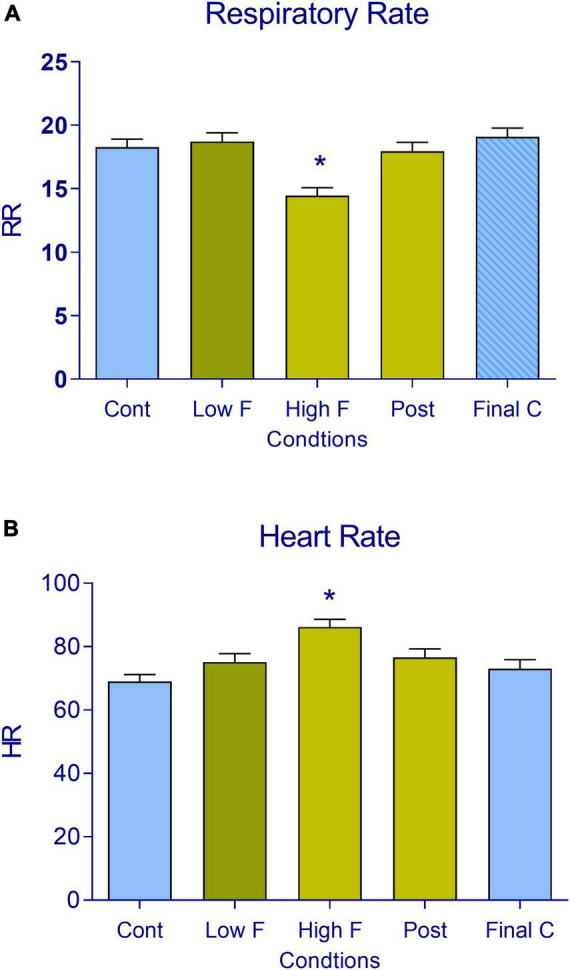
**(A)** Respiratory rate and **(B)** heart rate in subjects during the virtual reality task. The data are expressed as mean + SEM. *n* = 22 across the five conditions (control, low fear, high fear, post-encounter, and final control). *p < 0.05, compared with the initial control.

For the analysis of respiratory rate and heart rate, we first examined these responses during the five phases of the test (Figures 3A,B, respectively). We found significant differences in respiratory rate [*F*(4, 84) = 15.41, *p* < 0.05] and heart rate [*F*(4, 84) = 16.72, *p* < 0.05] among these conditions. The Tukey multiple comparison analysis revealed decreases in respiratory rate and increases in heart rate in the high predator condition (*p* < 0.05). Proximal threat elicited a low respiratory rate and high heart rate, whereas distal threat was not of sufficient intensity to change these autonomic responses (see Supplementary Material).

We next investigated whether there was a relationship between types of threat and blood oxygen levels. We found no significant differences in blood oxygen levels among all conditions [*F*(4, 84) = 0.89, *p* > 0.05]. The level of threat that was used in the present study was associated with the maintenance of basal SpO_2_ levels. These results are reasonable when considering the consistent increase in blood oxygenation level-dependent responses in rostral and caudal brain regions during the AI high predator condition (10).

**FIGURE 4:**
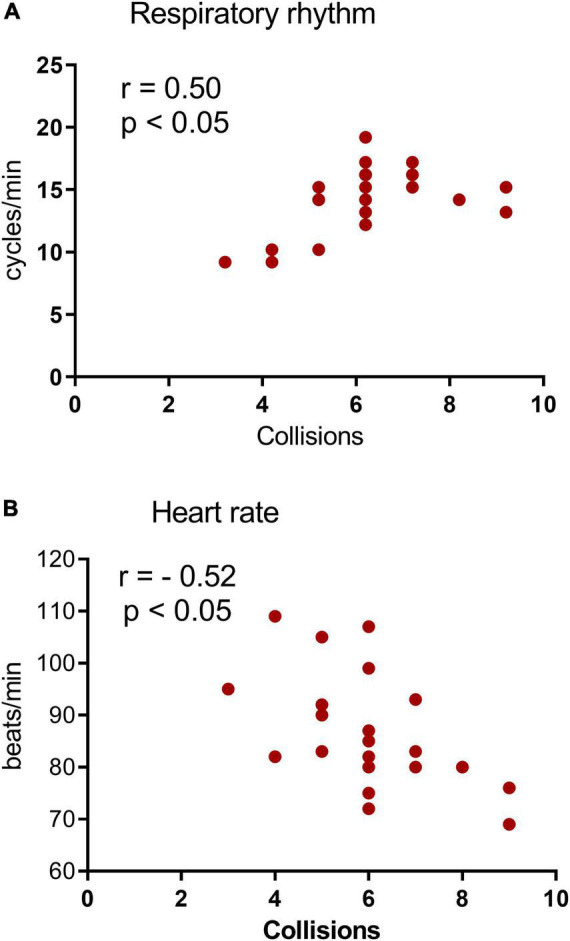
Subject-specific differences in respiratory rate (RR) and heart rate (HR) in the high fear condition (phase 3) of the VRP. **(A,B)** Scatterplots of RR **(A)** and HR **(B)** correlated with collisions in the high predator condition. Each point represents the recorded RR or HR in phase 3 of the VRP from each subject. *n* = 22. The number of points is less than the actual number of subjects because of several overlaps.

If the threat circuit is activated by distance and intensity factors that are related to the predator encounter, revealed by significant differences in avoidance responses among conditions, then we would expect specific indices of respiratory rate and heart rate of the proximal threat to correlate with autonomic indices of high fear. Indeed, the decrease in respiratory rate and increase in heart rate were both associated with the high predator condition (Pearson’s correlation coefficient: *r* = 0.50 for respiratory rate, *r* = 0.52 for heart rate; both *p* < 0.05). These effects are illustrated in Figure 4. Collisions did not correlate with changes in these autonomic parameters in the low fear condition (see Supplementary Figure 4).

## Discussion

Adaptations to threat are thought to be mainly organized along a single dimension of “predatory imminence.” Given the many levels of threat-danger, strategies that are adopted by individuals range from the pre-encounter global organization of behavior at low levels of threat to post-encounter avoidance and circa-strike defensive tactics when contact with the predator is highly likely (2, 13, 23–27). The results showed a significant reduction in the number of times the subjects were captured in the high predator phase (85 dB) vs. control phases, suggesting that the subjects were motivated to avoid the high predator. The present results align with Mobbs et al. (10, 11), in which they reported higher avoidance behavior when encountering a predator in the AI high fear condition. Altogether, these results suggest that subjects were more efficient in mlovement planning and execution before the encounter (i.e., avoidance behavior) than when escaping the predator since the AI prey is programmed after going outside the open field to randomly return anywhere in the open field (22 cm × 24 cm rectangle of walls), even next to the AI predator. The present findings indicated a significant correlation between VRP scores of the proximal threat and respiratory and heart rates. Although non-significant, post-encounter activity was evaluated here in the context of consolidation of post-stress memories triggered by avoidance behavior and to detect an eventual nocebo effect. Thus, the high fear encounter session and post-encounter session both have light green backgrounds but only the high fear session had loud noise (85 dB). Also, increasing the intertrial periods between the sessions in future studies could give a clue on the period necessary for the development of post-traumatic memories.

We tested the hypothesis that autonomic responses in humans who encounter distal or proximal threats mirror those that have been derived from rodent defense system models. We predicted that changes in respiratory rate and heart rate would depend on the distance or intensity of the threat, which is known to underlie different types of anxiety. To test this hypothesis, we examined defense reactions in subjects while they performed an active “avoidance/escape loud noise” task in a two-dimensional open field. In particular, the present study sought to associate behavioral responses to distal and proximal threats with respiration. Respiratory rate may be a useful candidate for bridging the responses of the brain and body during emotion and is an important physiological marker of an individual’s emotional state and modulates activity in a wide range of brain regions (22, 28–32). During emotional states, the body undergoes multiple changes that have been suggested to feedback to the brain to influence neural processing. The VRP that was used in the present study elicited a particular defense reaction that was characterized by a decrease in the number of times the subjects were captured in the high predator condition, a decrease in respiratory rate, an increase in heart rate, and the maintenance of SpO_2_ levels. This defense reaction pattern may be interpreted in the context of defensive strategies that are related to different levels of fear/anxiety, particularly when the paradigm categorizes defensive behaviors according to auditory threats and relates them specifically to their distance from the source of predatory imminence. As expected, heart rate (HR) increased along with active avoidance behavior. The increase in blood flow to supply oxygen to the muscles and the organism during fear states has been suggestive of anticipatory anxiety.

In this study, we give particular attention to respiration changes occurring during moderate and intense fear states generated by a new VRP model. In other words, instead of looking at physiological state in general (respiration, heart rate, and SpO_2_) this study is concerned with specific fear states. Animal studies of anxiety have shown an oscillatory state of breathing circuits that is entrained by 4 Hz in the olfactory bulb during fear states (22, 33). Using a classic auditory cue conditioning paradigm (tones-CS), 24 h after conditioning, rodents breathed irregularly during the active exploration of an environment (pre-tone), and respiratory rates rapidly alternated between sniffing bouts (6–12 Hz) and slow breathing. In contrast, during periods of freezing in the presence of tones (i.e., conditioned stimuli), respiratory rates became very steady, with little cycle-to-cycle variability and an average of approximately 4 Hz (22). In a study that used a similar experimental design, the respiratory frequency dropped, and tidal volume increased during freezing periods (33). These studies suggest that during freezing, breathing shifts to a lower, deeper, and more regular rhythm. Our results support this possibility, in which high predatory imminence in our model significantly decreased respiratory rate to lower levels. The precise nature of this bodily feedback and mechanisms that underlie its eventual impact on neural processing remains to be determined. Nonetheless, evidence suggests that the 4-Hz respiratory rate in the olfactory bulb is a crucial regulator of brain states during stressful situations (34).

The neural substrates of fear in the inferior colliculus respond to increasing noise intensities, sending ascending information to the medial geniculate nucleus of the thalamus and then to the amygdala (3, 5). Indeed, the stimulation of these areas can initiate freezing behavior, and these structures are known to modify breathing, possibly via the nucleus retroambiguus (21). Thus, depending on the intensity and nature of aversive stimuli to which an individual is exposed, the pattern of defensive reactions changes. We presume that respiration-related rate triggers a cognitive component of the defense reaction that is relevant for separating fear, anxiety, and panic-like states. Our findings may have implications for revealing the neurobiology of emotional disturbances in the face of a threat that is posed by different types of aversive stimuli. Indeed, recognizing emotional stimuli has been shown in most circumstances to be processed outside of conscious awareness and result in increases in heart and respiratory rates. However, in some cases, these results cannot be generalized and may depend on the individual’s cognitive state (35). Indeed, as commented earlier the individual’s cognitive state has to be considered to explain the correlations obtained in the analysis of the present results. Among all physical emotional reactions to threats, respiration is unique in that people may voluntarily influence their emotional state by changing their respiration patterns.

A prominent theory that associates gas level changes with anxiety is the suffocation false alarm model. This model predicts that spontaneous panic attacks occur when the brainstem misreads signals that are linked to changes in O_2_/CO_2_ and activates a maladaptive autonomic response to perceived suffocation (36). Either an increase in CO_2_ levels or decrease in O_2_ levels can trigger anxiety- or panic-like feelings. In the present study, we found no changes in O_2_ saturation in blood, suggesting that our experimental conditions did not elicit autonomic responses that are evident in a false alarm model.

Finally, the present study evaluated behavioral, autonomic, and SpO_2_ effects of the distance and intensity of a threat that was associated with proximal danger in adult human volunteers during a dynamic avoidance threat-of-loud noise paradigm. Our findings contribute to previous empirical data on individual profiles of defense reactions, establishing defense reaction patterns that may serve as a reference for characterizing an individual’s emotional susceptibility to the development of distinct types of anxiety. Our use of a predatory imminence paradigm may be a step toward further large-scale studies of overall defense reaction patterns that precede the development of anxiety and may be useful for refining diagnoses of distinct types of anxiety. The data obtained in the low-fear, high-fear, and post-encounter phases in the present study may be useful for anticipating the degree of emotional reactivity of individuals who undergo this test, providing some clues about their state or trait anxiety. Further studies with large samples that use well-controlled tasks and advanced data acquisition, and processing techniques will bolster the robustness and relevance of our findings, especially concerning establishing new methods that can detect individual vulnerability to anxiety disorders.

## Data Availability Statement

The original contributions presented in the study are included in the article/Supplementary Material, further inquiries can be directed to the corresponding author/s.

## Ethics Statement

The studies involving human participants were reviewed and approved by the University of São Paulo Institutional Review Board (no. 51983721.0.0000.5407). The patients/participants provided their written informed consent to participate in this study.

## Author Contributions

MB performed the conception and design of the study, data analysis, and drafted the manuscript. MN and RE collected the preliminary data, provided critical revisions, and approved the final version of the manuscript for submission. All authors contributed to the article and approved the submitted version.

## Conflict of Interest

The authors declare that the research was conducted in the absence of any commercial or financial relationships that could be construed as a potential conflict of interest.

## Publisher’s Note

All claims expressed in this article are solely those of the authors and do not necessarily represent those of their affiliated organizations, or those of the publisher, the editors and the reviewers. Any product that may be evaluated in this article, or claim that may be made by its manufacturer, is not guaranteed or endorsed by the publisher.

## Footnotes

1
http://www.python.org

2
www.novaknup.com.br

3
www.graphpad.com
