# Supplementary material for: Testing Emotional Vulnerability to Threat in Adults Using a Virtual Reality Paradigm of Fear Associated With Autonomic Variables
Source: Front Psychiatry. 2022 Apr 1;13:860447. doi: 10.3389/fpsyt.2022.860447 (PMC9010675; doi:10.3389/fpsyt.2022.860447)
Supplement: Supplementary file 1 [file Data_Sheet_1.pdf]

Supplemental Material

Testing emotional vulnerability to threat in adults using a virtual reality paradigm of fear associated with autonomic variables

Marcus L Brandão,[1, 2,] * Manoel Jorge Nobre,[1] Ruth Estevão,[1]

[1] Instituto de Neurociências e Comportamento, Avenida do Café, 2450, Ribeirão Preto, São Paulo, 14050-220, Brazil

[2] NAP-USP-Neurobiology of Emotions Research Centre (NuPNE), Ribeirão Preto Medical School, University of São Paulo (FMRP-USP), Av. Bandeirantes, 3900, Ribeirão Preto, São Paulo, 14049-900, Brazil.

Running title: stress vulnerability

Keywords: Fear, Anxiety, Emotional resilience

* Correspondence: Instituto de Neurociências e Comportamento, Avenida do Café, 2450, Ribeirão Preto, São Paulo, 14050-220, Brazil. email: mbrandao@usp.br

This file includes:
Materials and Methods
Fig. S1 to S4
Table S1

Methods and Materials

Subjects

We tested 24 healthy subjects. All were Portuguese-speaking and right-handed, had normal or corrected-to-normal vision, and were screened for a history of psychiatric and neurological disorders. All subjects gave written informed consent. The study was approved by the joint Ethics Committee of Platform Brazil and the Faculty of Medicine of Ribeirão Preto, University of São Paulo. Two subjects were not analyzed because of technical problems with data acquisition, leaving a total of 22 subjects (mean age ± standard deviation: 48.50 ± 12.72 years). Figure S1 illustrates a subject performing the virtual reality paradigm.

Experimental procedures

Sound calibration. Sound (white noise) was previously taken at the desired frequency from the NCH software library. The intensity of the sound was tested before the experiment and set to 80- and 85-dB using a (KP 8015, Knup, São Paulo, Brazil; www.novaknup.com.br) decibel meter.

Artificial intelligence program. A recursive template (1) was implemented to control the behavior of the artificial intelligence (AI) predator (red rectangle). All valid adjacent positions (i.e., not outside the lines that delimited the open field rectangle) from the current position (maximum of 4) were considered for the next movement, with the collision with the blue rectangle (prey) computed for each. Movements outside the valid rectangle that delimited the open field were considered an escape response, signaled by a beep tone. The AI predator was programmed to be slightly faster than the subject's calibrated speed. The session comprised five 60-s blocks, each with a particular background color to define the condition (Figure S2). To

dissociate spatial and temporal elements of imminence, the position of the AI randomly changed every time it collided with the prey.

Subjects used a keypad to move the blue triangle and were given time to practice the task both in and out of the open field. To reduce any motion and confounds that were caused by repetitive key presses, the blue triangle could be moved by continuously holding the keys.

Paradigm. Subjects were presented with a two-dimensional open field that contained a 22 cm x 24 cm rectangle (dark blue). Based on ecological models of predator and prey interactions, the paradigm consisted of five phases, each signaled by a change in the background color. In all phases, subjects moved the blue rectangle to escape the AI predator. During the neutral phase (gray background), subjects were informed of the amount of aversive noise they would receive if the AI predator captured them: 80 dB (AI low) or 85 dB (AI high). The AI was programmed to wander the open field indiscriminately. All experimental conditions commenced with a neutral phase where a pre-programmed AI symbol appeared at the upper-left side of the maze. Afterward, the control phase commenced, during which the AI changed into a predator. The switch to the blue background indicated to the subject that they would view the control condition with no consequences. In the next two phases, the background color changed to green and light green, signaling that each predator/prey encounter would result in the emission of 80- or 85-dB white noise (low and high fear phases, respectively). There were two additional conditions in the experimental session: one post-encounter phase that was similar to the high fear phase but with a bell tone instead of white noise and a final control phase (blue background) to determine whether there were any residual effects after the post-encounter phase. The whole session lasted approximately 6 min (Figure S2)

Behavioral recordings. We recorded the number of collisions and escape responses by the subject in the open field in each phase of the test (Figure S3A).

Autonomic responses. Simultaneously with the virtual reality paradigm, a belt coupled to a respiratory rate sensor was lightly fastened at the mid-thorax of the subject. Once seated in front of the computer, an oximeter was placed on the left index finger. Both the oximeter and respiratory rate sensor were connected to an interface that was programmed to monitor vital signs (Insight Equipment, Ribeirão Preto, São Paulo, Brazil), including respiratory rate, heart rate, and SpO_2 levels. (Figure S3B).

Correlations. Individual differences of the interactions between avoidance behavior and autonomic activity. Avoidance behavior did not correlate with heart rate (A) and respiratory rate (B) in the AI low fear condition (Figure S4)

Figure S1. Illustration of a female subject performing the virtual reality paradigm. Two computers were set for running the experiments. One ran the VRP developed at the Institute of Neurosciences and Behavior (INeC) and the other ran the program for recordings of the autonomic measures (Insight Equipments). Both programs were synchronized by a domestic network so that both result outcomes could be stored in Excel spreadsheets for later analysis.

Figure S2. **Time schedule of the virtual task, showing an illustration of the paradigm schedule.** Initially, there was a 60 s familiarization phase, during which the participants were presented with the main aspects of the task. Each experimental session included five phases: familiarization (gray), control (light blue), low fear (green), high fear (light green), post-encounter (light green), and final control (light blue). In the initial and final control and post-encounter phases, no white noise was delivered, but the subjects could move the blue rectangle to avoid encountering the predator. The fear phase began once the aversive stimuli could be delivered, depending on whether the condition was low fear (80 dB white noise) or high fear (85 dB white noise). The post-encounter phase was like the fear phase, with the exception that there was no white noise emitted, which was replaced by a bell tone. Each 60 s phase terminated with a frozen screen for 5 s. The noises were emitted only when there was contact with the AI during the encounter phase, signaled by a green (low fear) or light green (high fear) background. The noise was applied for 0.5 s after contact.

Figure S3. (**Left**) Illustration of prey avoidance behavior (blue rectangle) from the predator (red rectangle) in the virtual reality paradigm. (**Right**) Recording of autonomic responses during a subject's performance during the low-fear phase of the virtual reality paradigm. (**Top**) Respiratory plethysmography was recorded in conjunction with heart rate and SPO_2.

Figure S4. Scatterplots of (**A**) respiratory rate and (**B**) heart rate correlated with the number of prey/predator collisions during the virtual reality paradigm according to Pearson's correlation coefficients. Each point represents the recorded RR or HR of an individual's response in phase 2 of the virtual reality paradigm (low fear). The number of points is less than the actual number of subjects because of some overlaps.

References

De Oliveira AR, Colombo AC, Muthuraju S, Almada RC, Brandão M (2014): Dopamine D2-like receptors modulate unconditioned fear: role of the inferior colliculus. PLoS One 9:e104228.

Mobbs D, Petrovic P, Marchant JL, Hassabis D, Weiskopf N, Seymour B, Dolan RJ, Frith CD (2007): When fear is near: threat imminence elicits prefrontal–periaqueductal gray shifts in humans. *Science* 317:1079-1083.

Petiot JC, J Parrot J, Lobreaul JP, Smolik HJ (1992): Cardiovascular effects of impulse noise, road traffic noise, and intermittent pink noise at LAeq = 75 d B, as a function of sex, age, and level of anxiety: a comparative study. II. Digital pulse level and blood pressure data. *Int Arch Occup Environ Health* 63:485-493.

Reimer AE, Oliveira AR, Brandão ML (2008): Selective involvement of GABAergic mechanisms of the dorsal periaqueductal gray and inferior colliculus on the memory of the contextual fear as assessed by the fear-potentiated startle test. *Brain Res Bull* 76:545-550.

Rodríguez-Arce J, Lara-Flores L, Portillo-Rodríguez O, Martínez-Méndez R (2020): Towards an anxiety and stress recognition system for academic environments based on physiological features. *Comput Methods Programs Biomed* 190:105408.

Russell SJ. Norvig P (2021): Artificial intelligence: a modern approach, 4th edition. Harlow: Pearson Education.

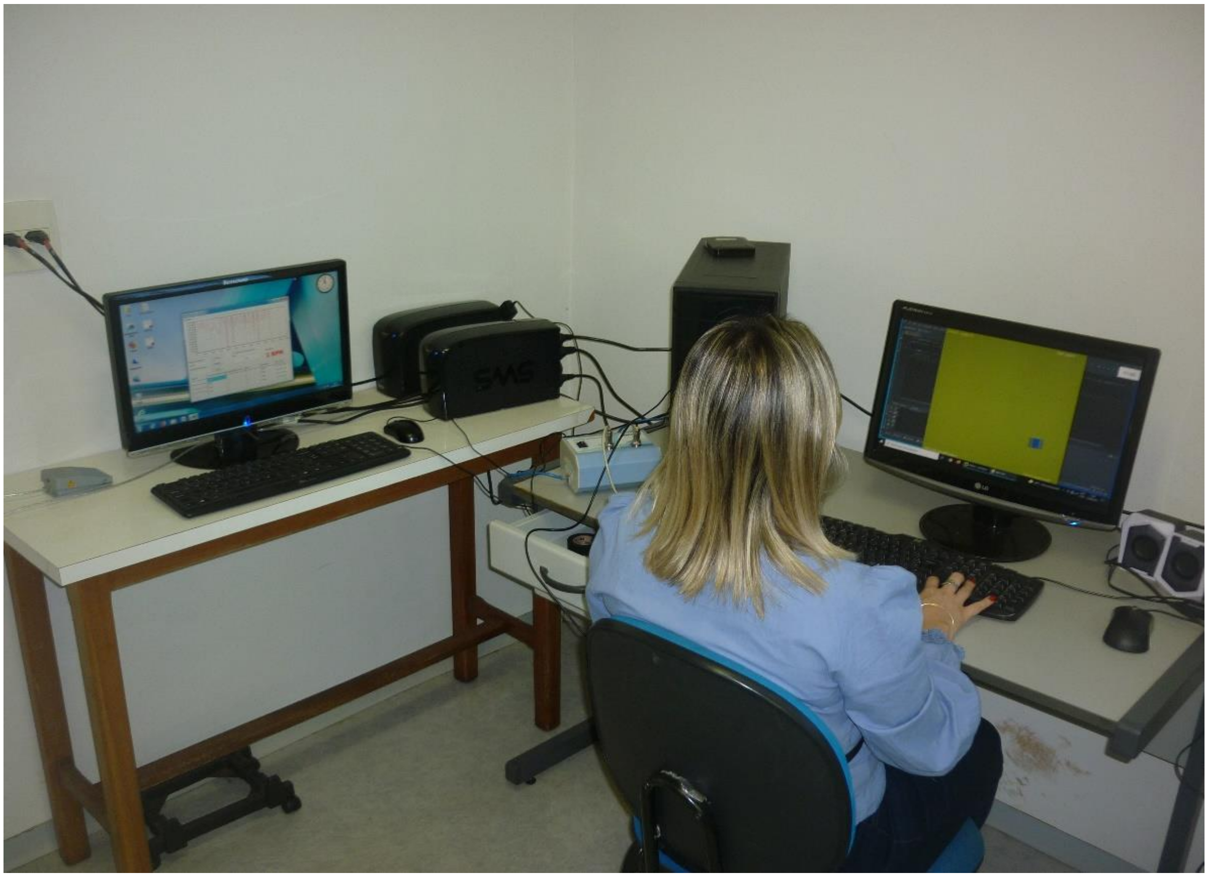

Figure S1

Experimental design

1. Neutral phase	2. Pre-encounter	3. Low fear encounter	4. High fear encounter	5. Post-encounter (same context without white noise)	6. Post-encounter control
Training of prey (blue rectangle) to avoid predator (red rectangle). Collision produces a ding tone.	Session in which predator and prey collision produces a bell tone. Escape from the open field causes a computer tone.	Session in which collision of predator and prey causes a 0.5 s white noise of 80 dB.	Similar session in which collision of predator and prey causes a 0.5 s white noise of 85 dB.	Similar session in which collision of predator and prey causes a bell tone.	Session similar to pre-encounter
Gray background	Blue background	Green background	Light green background	Light green background	Blue background

Figure S2

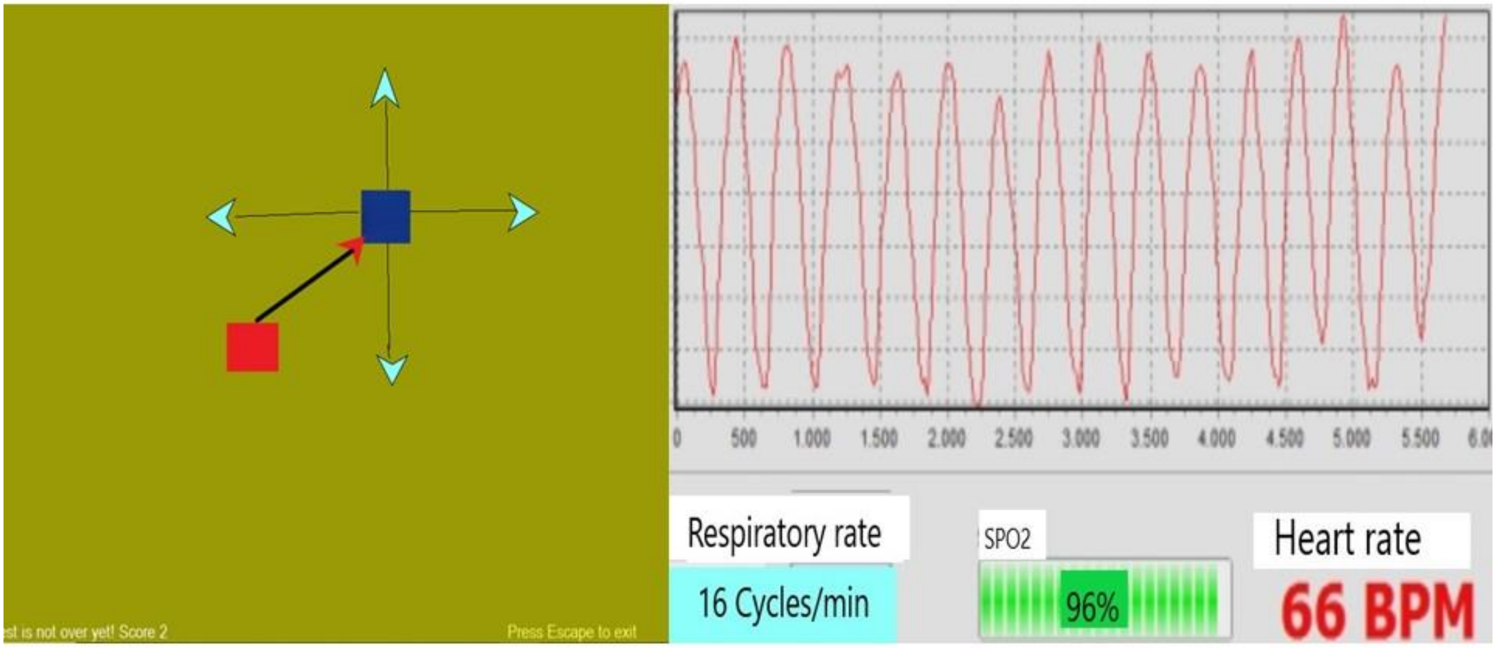

est is not over yet! Score 2
Press Escape to exit
0
500
1.000
1.500
2.000
2.500
3.000
3.500
4.000
4.500
5.000
5.500
Respiratory rate
16 Cycles/min
SPO2
96%
Heart rate
66 BPM

Figure S3

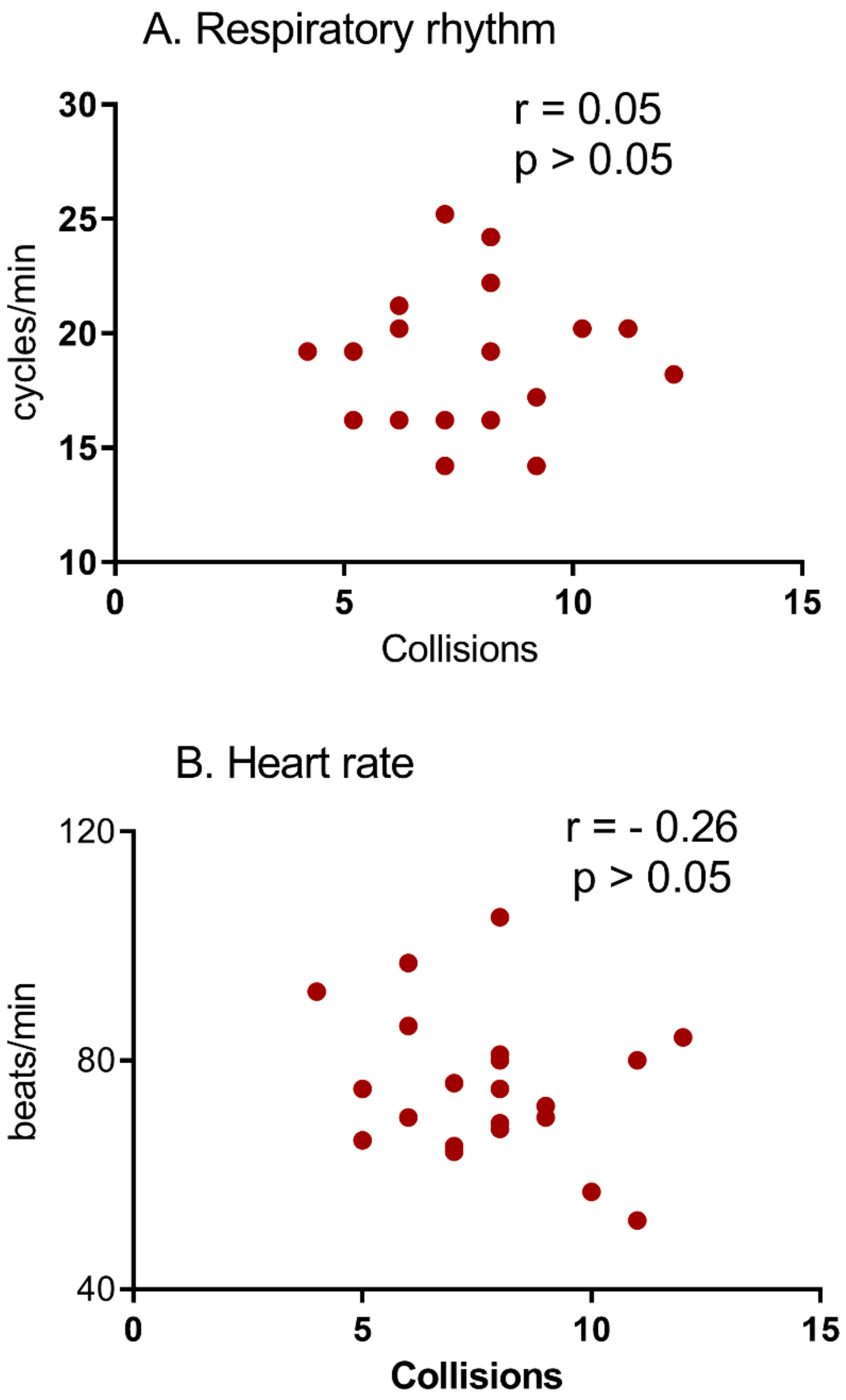
A. Respiratory rhythm
r = 0.05
p > 0.05
cycles/min
30
25
20
15
10
0
5
10
15
Collisions
B. Heart rate
r = - 0.26
p > 0.05
beats/min
120
80
40
0
5
10
15
Collisions

Figure S4

Supplementary Table

Table S1 – Demographical data of subjects

	Males	Females
**Age (SD)**	50.1(10.9)	46.6(13.8)
**History of mental disorders**	No	No
**Race**		
**White**	5	3
**Brown**	7	7
**Education**		
**High**	7	7
**Medium**	5	3
**History Covid- 19**	No	No
**Baseline blood pressure**	Normal	Normal
